# Cell Timer/Cell Clock

**Published:** 2018-11

**Authors:** Hamed Ghadiri, Sana Alavi, Bahareh Dabirmanesh, Kenji Moriyama, Khosro Khajeh, Hisao Masai

**Affiliations:** 1 Faculty of Biological Sciences, Tarbiat Modares University, Tehran 14115-175, Iran; 2 Department of Genome Medicine, Tokyo Metropolitan Institute of Medical Science, 2-1-6 Kamkitazawa, Setagaya-ku, Tokyo 156-8506, Japan

## Like the biological clock in the body, replication of each cell type (even different cells of the same organism) follows a timing program. Abnormal function of this timer could be an alarm for a disease like cancer.

DNA replication starts from a specific point on the chromosome that is called the origin of replication. In contrast to prokaryotes in which DNA replication starts from a single origin, eukaryotic DNA replication starts from many origins scattered along the chromosomes. Budding yeast contains 300 origins, whereas fission yeast has 1,100, and the numbers of replication origins for human increase to over 20,000. These origins are fired in a coordinated manner, and there are spatial and temporal disciplines for this process, which happens in the S phase of the cell cycle. It was known that eukaryotic cells prepare all these potential origins during the G1 phase of the cell cycle but utilize only a portion of these origins during S phase. Furthermore, firing some of these origins are delayed until the mid and late phases of the S phase. Coordinated activation of these origins occurs under “Replication Timing Program”. The segments of the chromosome containing co-regulated origins that fire simultaneously are named “Replication Timing Domains”, ranging in size from 100 kb to 1 Mb. Replication timing is determined at a specific time in the early G1 phase that is called Timing Decision Point (TDP). Studies have shown that major chromosome repositioning occurs at TDP. Generally, replication timing domains are classified into three classes including Early, Mid and Late.

## Identification of the Early and Late origins

There are three suggested mechanisms by which cell can distinguish the Early and other types of replication origins.

First mechanism: The origins of replication are marked by the pre-RC components through specific covalent modification.

Second mechanism: Firing of Mid/Late origins is prevented by Rif1 via two mechanisms. The first is done by recruiting phosphatase that inhibits the firing of origins. The second is carried out by creating chromatin structures that represent replication-suppressive domains.

Third mechanism: Early firing is promoted by open chromatin structure or by euchromatic marks. Fkh1 is a protein known to recruit Cdc7 kinase, specifically to early-firing origins.

## Rif1 protein as a chromatin architect

Recent studies have found many factors regulating the replication timing. One of the most important factors is the Rif1 protein that plays a key role in the regulation of replication timing in yeast and higher eukaryotes. Rif1 protein may regulate the replication timing domains by organizing the chromatin loop structures. A C-terminal domain of Rif1 is able to bind DNA on its own and also become oligomerized. Through its ability to hold many chromatin fibers together by the DNA binding and oligomerization activities, C-terminal domain of Rif1 creates chromatin compartments that are inhibitory for origin firing. Rif1 capability to regulate the chromatin architecture and consequently to regulate the DNA replication timing could have versatile impacts on other chromosome functions, including recombination, transcription, and repair.

## Replication timing and disease

Replication timing is amongst the cellular phenomena being modified during some diseases including cancer (especially breast cancer). The deregulation of the replication timing has been demonstrated to result in chromosomal disorders and genomic instabilities. More importantly, cellular changes that can cause tumors are accompanied by replication timing program disturbances. These observations could lead to a suggestion that Rif1, one of the key factors in replication timing, could be a diagnostic biomarker or a drug target for various malignancies. Studies have shown that changes in the replication timing are not limited to cancer but are also linked to some of the inheritable diseases

Overall, considering the multifunctional nature of Rif1 and its connection with some diseases including cancer, the design and set up of a platform for full-length Rif1 expression or each of its domain seems to be necessitated. At the same time, analysis of its interaction with DNA and other cellular proteins may provide a suitable target for future practical studies like drug discovery.

**More details in:**

1. ***Anatomy of mammalian replication domains***. S Takebayashi et al. 2017. MDPI; Vol. 8, 1-12.
2. ***Temporal and spatial regulation of eukaryotic DNA replication: From regulated initiation to genome-scale timing program***. C Renard-guillet et al. 2014. Semin Cell Dev Biol; Vol. 30, 110-120.
3. ***DNA replication timing influences gene expression level***. CA Müller et al. 2012; J Cell Biol; 1-8.
4. ***DNA replication origin activation in space and time***. M Fragkos et al. 2015. Nature; Vol. 16, 360-374.
5. ***Rif1 times replication replication***. P Strzyz. 2015. Nat Rev Mol Cell Biol 2016; Vol. 17, 2662.
6. ***A conserved chromatin factor regulating DNA replication, DNA repair, and transcription***. ***In: The Initiation of DNA Replication in Eukaryotes Springer International Publishing*.** N Yoshizawa-Sugata et al. 2016. pp. 143-158.
7. ***Forkhead transcription factors establish origin timing and long-range clustering in S. cerevisiae***. SR Knott et al. 2012. Cell; Vol. 148, 99-111.
8. ***Replication timing regulation of eukaryotic replicons: Rif1 as a global regulator of replication timing*.** S Yamazaki et al. 2013. Trends Genet; Vol. 29, 449-460.
9. ***Alterations in replication timing of cancer-related genes in malignant human breast cancer cells***. Fritz AJ. 2013. J Cell Biochem; Vol. 114, 1074-1083.
10. ***DNA replication timing, genome stability and cancer*.** N Donley et al. 2013. Semin Cancer Biol; Vol. 23, 80-89.
11. ***Alterations in replication timing of cancer related genes in malignant human breast cancer cells*.** AJ Fritz et al. 2013. J Cell Biochem; Vol. 114, 1074-1083.
12. ***Perturbations in the replication program contribute to genomic instability in cancer***. B Blumenfeld B et al. 2017. Int J Mol Sci; Vol. 18, 1138.
13. ***Abnormal developmental control of replication-timing domains in pediatric acute lymphoblastic leukemia***. T Ryba T et al. 2012. Genome Res; Vol. 22, 1833-1844.
14. ***Human RIF1 encodes an anti-apoptotic factor required for DNA repair***. H Wang et al. 2009. Carcinogenesis; Vol. 30, 1314-1319.
15. ***Epigenetic abnormalities associated with a chromosome 18(q21-q22) inversion and a Gilles de la Tourette syndrome phenotype*.** MW State et al. 2003. Proc Natl Acad Sci USA; Vol. 100, 4684-4689.
16. ***Altered replication timing of the HIRA/ Tuple1 locus in the DiGeorge and velocardiofacial syndromes***. S D’Antoni et al. 2004. Gene; Vol. 333, 111-119.
17. ***Hypomethylation of subtelomeric regions in ICF syndrome is associated with abnormally short telomeres and enhanced transcription from telomeric regions***. S Yehezkel et al. 2008. Hum Mol Genet; Vol. 17, 2776-2789.

